# Correction of confidence intervals in excess relative risk models using Monte Carlo dosimetry systems with shared errors

**DOI:** 10.1371/journal.pone.0174641

**Published:** 2017-04-03

**Authors:** Zhuo Zhang, Dale L. Preston, Mikhail Sokolnikov, Bruce A. Napier, Marina Degteva, Brian Moroz, Vadim Vostrotin, Elena Shiskina, Alan Birchall, Daniel O. Stram

**Affiliations:** 1 Keck School of Medicine, University of Southern California, Los Angeles, CA, United States of America; 2 Hirosoft International Corporation, Eureka, CA, United States of America; 3 Southern Urals Biophysics Institute, Ozersk, Russia; 4 Pacific Northwest National Laboratory, Richland, WA, United States of America; 5 Urals Research Center for Radiation Medicine, Chelyabinsk, Russia; 6 Global Dosimetry Limited, Didcot, Oxon, United Kingdom; University of North Carolina at Chapel Hill, UNITED STATES

## Abstract

In epidemiological studies, exposures of interest are often measured with uncertainties, which may be independent or correlated. Independent errors can often be characterized relatively easily while correlated measurement errors have shared and hierarchical components that complicate the description of their structure. For some important studies, Monte Carlo dosimetry systems that provide multiple realizations of exposure estimates have been used to represent such complex error structures. While the effects of independent measurement errors on parameter estimation and methods to correct these effects have been studied comprehensively in the epidemiological literature, the literature on the effects of correlated errors, and associated correction methods is much more sparse. In this paper, we implement a novel method that calculates corrected confidence intervals based on the approximate asymptotic distribution of parameter estimates in linear excess relative risk (ERR) models. These models are widely used in survival analysis, particularly in radiation epidemiology. Specifically, for the dose effect estimate of interest (increase in relative risk per unit dose), a mixture distribution consisting of a normal and a lognormal component is applied. This choice of asymptotic approximation guarantees that corrected confidence intervals will always be bounded, a result which does not hold under a normal approximation. A simulation study was conducted to evaluate the proposed method in survival analysis using a realistic ERR model. We used both simulated Monte Carlo dosimetry systems (MCDS) and actual dose histories from the Mayak Worker Dosimetry System 2013, a MCDS for plutonium exposures in the Mayak Worker Cohort. Results show our proposed methods provide much improved coverage probabilities for the dose effect parameter, and noticeable improvements for other model parameters.

## Introduction

Measurement errors are ubiquitous in epidemiological studies, especially for doses arising from environmental and occupational exposures, which are difficult to assess. These errors may cause problems in risk estimation and statistical inference, possibly leading to incorrect conclusions [1]. The most well-known measurement error models are classical and Berkson. In linear models, classical measurement errors change the mean structure and thus introduce bias in parameter estimates, while averaging errors only change variances without biasing parameter estimates. Correction methods for independent measurement errors have been comprehensively described elsewhere [2], including regression calibration, simulation extrapolation (SIMEX), etc. However, when exposure estimates in a cohort are constructed using complicated physical and biological models, uncertainties in the dosimetry system can become very complex [3]. In such systems, uncertainties of some parameters in the models may affect a large group of study participants simultaneously.

In radiation epidemiology, there are a number of important studies for which dosimetrists have developed Monte Carlo dosimetry systems (MCDS) [4–6] to represent the structure of uncertainties in dose estimation. Samples from MCDS are given as dose realizations/replications, which provide both dose estimates and associated uncertainty information, in contrast to traditional dosimetry systems where only a single point estimate is given. Usually there are many dose determining parameters that are shared by sets of individuals in MCDS. These shared parameters are sampled and fixed in each dose replication. Thus, dose replications can preserve the correlation structure of dose errors. One example is CIDER (Calculation of Individual Doses from Environmental Radionuclides) dosimetry system that models exposure of the thyroid gland to radioactive iodine (^131^I), used in Hanford Thyroid Disease study [7]. In this system, shared components with uncertainties included the total release of ^131^I from the site, prevailing weather patterns, pasturing practices, biological parameters affecting uptake to the thyroid gland, etc. To represent these uncertainties, CIDER system provided 100 dose replications for the entire cohort, where both shared and unshared components affect the correlation structure of the replications. Other examples include studies of ^131^I exposure in Kazakhstan [8], and studies of occupational and residential exposure to radiation from the Mayak plutonium production plant in the Southern Urals region of Russia. We are particularly motivated by work on dosimetry for the Techa River Cohort study [9–11] and the Mayak Workers Cohort study [3, 12]. The dosimetry system for the Mayak Workers Cohort study is used as the basis of our discussion and the simulation experiments described below.

In order to study the effects of shared uncertainties on epidemiologic risk estimation when a MCDS is available, Stram and Kopecky [13] proposed the shared and unshared, multiplicative and additive (SUMA) model to capture some aspects of a complex dosimetry system represented by Monte Carlo replications. Stram et al. [14] proposed using the mean dose, obtained by averaging across all replications, in place of the true dose in risk analysis and described a sandwich estimation technique for correcting the variance calculation of the regression model parameter estimates. The authors claimed that by using the mean dose, the score estimation equation has expected value zero at the true parameter values, which keeps the parameter estimator unbiased. With their approach, the authors performed a score-type test for inference in an example. However, the construction of confidence interval (CI) is not specified and CI’s constructed based on the score-type test can be unbounded. Alternatively, Kwon [15] proposed a Bayesian approach that involves calculating the likelihood for each dose replication and averaging the posterior samples of model parameters fitted to each dose replication weighted by their associated likelihood values. The authors claimed their approach greatly improved coverage probability and reduced bias. We note, however, this appears to be in contradiction to previous conclusions regarding simultaneous estimation of true dose along with the model parameters; see section 7.1 of [2].

In this paper, we propose a novel approach to approximate the asymptotic distribution of parameter estimates in Poisson excess relative risk (ERR) models, using an MCDS. We also show how to construct CI’s based on this approximation, which can equivalently be used for hypothesis testing. Specifically, for the dose effect parameter, we use a mixture of normal and lognormal distributions to approximate its distribution, rather than the normal distribution as commonly used in maximum likelihood theory. The variance of the two components in this mixture distribution are estimated using techniques as in [14]. Based on this mixture distribution, we calculate a score-type CI. A simulation study was performed to evaluate the performance of our method, compared with other procedures. The simulations used hypothetical SUMA dosimetry systems with various error settings, and also the Mayak Worker Dosimetry System 2013 (MWDS-2013), a newly-developed MCDS for the Mayak Worker Cohort [16]. Implementation of the proposed method is provided in R [17], as a package available online (https://github.com/zhuozhang/Rerr).

## Data and methods

### Mayak worker cohort and the dosimetry system

The Mayak Production Association (Mayak PA) was established in 1948 as a facility for plutonium production in the former Soviet Union, located in the Southern Urals of Russia [18]. Due to inadequate knowledge of the radioactive materials, the workers in Mayak PA experienced chronic external and internal exposure to radiation through the following decades with especially high rates during the first decade of operation. The average cumulative external doses of the cohort, largely from gamma rays, were larger than the average doses received by Japanese atomic bomb survivors in the Life Span Study [19] and substantially larger than those received at similar nuclear production sites such as the Hanford facility [3]. Workers in the radiochemical and plutonium production plants had the potential for internal exposures due from inhaled plutonium aerosols at, on average, much higher levels than those experienced by US [20] or UK workers [21] engaged in similar work.

The Mayak Worker Cohort (MWC) was established in the 1980’s by Russian investigators. Support for the study of this cohort is currently provided by the Russian Ministry of Health and (through a bi-national cooperative agreement between Russia and the United States) from the U.S. Department of Energy. As currently defined, the cohort includes 25,757 people who began work at one of the main plants (reactor complex, radiochemical plant, or plutonium production plant) or in one of two auxiliary departments (water treatment or mechanical repair) between 1948 and 1982 [22]. About 25% of the cohort members are women. Estimates of individual annual occupational external doses to various organs are available for all cohort members. These estimates were largely based on individual film badge readings augmented with information about specific workplaces and job titles. Around 17,000 MWC members who worked in the radiochemical or plutonium production plants had potential for exposure to plutonium through the inhalation of plutonium-containing aerosols. About 8,000 of these workers (47%) have been measured for interval plutonium exposure using urine bioassays or from autopsy data. Doses to the lung, liver, bone surface, and various other organs have been computed for these monitored workers. Recent analyses have been based on the deterministic dose estimates provided by the Mayak Worker Dosimetry System 2008 (MWDS-2008) [23]. More recently, a Monte-Carlo dosimetry system has been developed for use in analyses of radiation risk in the MWC. The new system, called the Mayak Worker Dosimetry System 2013 (MWDS-2013), provides 500 realizations of annual external organ doses for each cohort member and 1,000 realizations of annual internal dose to various organs for cohort members with urinalysis or autopsy data.

Compared to other cohorts, the relatively high and protracted doses make the Mayak Worker Cohort extremely valuable for evaluating scientific hypothesis concerning dose rate and risk. The quality of death ascertainment, together with the availability of both internal and external radiation exposures, offers a unique opportunity to evaluate site-specific cancer mortality risks in both males and females under different exposure conditions that relates more closely to typical nuclear workers. It may also provide better characterization of occupational radiation risk both internally and externally.

The simulations described below made use of sex, date of birth, follow-up starting date and average of cumulative annual external dose for a subset of the MWC. This subset included 125,110 person-years of follow-up for 7,873 radiochemical and plutonium plant workers with plutonium bioassay data and internal dose estimates. As described in the following section, some of the simulations make use of the MWDS-2013 internal dose realizations for these workers.

### Poisson ERR model

Our methods and simulations are based on Poisson ERR models. These models are widely used for cancer risk analysis in radiation epidemiology. A reasonably general form for these ERR models is as follows,

$$h(t)=background(t)*\left(1+\sum_{i}b_{i}X_{i}(t)*{modifier}_{i}(t)\right),$$

$$background(t)=\exp\left(a_{0}+\sum_{j}a_{j}C_{j}(t)\right),$$

$${modifier}_{i}(t)=\exp\left(\sum_{k_{i}}a_{k_{i}}A_{k_{i}}(t)\right),$$

where *h*(*t*) is the hazard function as a function of time variable *t*, which can be age, or time since enrollment. The variable *X*_*i*_(*t*) represents time dependent exposure, e.g. cumulative internal or external radiation. The modifier_*i*_(*t*) term allows covariates, represented by $A_{k_{i}}(t)$, such as sex or time-dependent variables like age, to modify the risk associated with each *X*_*i*_(*t*). The background(*t*) term models the baseline risk applicable to the unexposed, generally as a function of both fixed covariates and time-dependent variables (e.g. age), notated as *C*_*j*_(*t*). We use time-dependent notations for all covariates without loss of generality, treating fixed variables as special cases of time-dependent variables.

Holford [24] and Laird and Oliver [25] observed the equivalence of performing survival analysis based on a piecewise exponential model and the log-linear Poisson model, by showing their likelihood functions only differ by a known constant factor. This equivalence holds generally for Poisson regression and survival analysis with other risk models, including the ERR model (see S1 File). In our simulations, we consider the following piecewise hazard model, which is only a slight simplification of models used for cancer risk analysis in the Mayak Workers Cohort,

$$\begin{array}{c} h_{i}=\exp\left(a_{0}+a_{1}C_{1,i}+a_{2}C_{2.i}+a_{3}C_{3,i}\right)(1+b_{1}X_{1,i}\exp{(a}_{4}A_{1,i}+a_{5}A_{2,i})+b_{2}X_{2,i}). \end{array}$$ #(1)

Here *i* indexes person-year (as in the following), and all covariates are described in Table 1.

**Table 1:** Description of variables in the hazard model.

*C*_1_	*C*_2_	*C*_3_	*X*_1_	*A*_1_	*A*_2_	*X*_2_
log(age/60)	log^2^(age/60)	sex	internal dose	log(age/60)	sex	external dose

Since internal dose estimates are considerably more uncertain than external dose estimates, here we assume only *X*_*1*_ is associated with measurement errors, with dose replications provided from a MCDS.

### A simplified model for Monte Carlo dosimetry systems

Stram and Kopecky [13] proposed a simplified model to characterize uncertainty components in a MCDS. This model includes four error components, namely shared and unshared multiplicative and additive (SUMA) errors. Let *X*_*i*_ be the true dose, and assume a Berkson error model, i.e., E(*X*_*i*_) = *Z*_*i*_, where *Z*_*i*_ is the measurement for *X*_*i*_. Under the SUMA model, the true dose *X*_*i*_ given the mean dose *Z*_*i*_ is represented as

$$X_{i}=\epsilon_{SM}\epsilon_{M,i}Z_{i}+\epsilon_{SA}+\epsilon_{A,i},$$

where subscript *S* denotes shared errors, subscript *M* denotes multiplicative errors, and subscript *A* denotes additive errors, with E(*ϵ*_*SM*_) = E(*ϵ*_*M*,*i*_) = 1 and E(*ϵ*_*SA*_) = E(*ϵ*_*A*,*i*_) = 0. While this model is oversimplified compared to an actual complex dosimetry system, it is useful for theoretical analyses of the effect of shared and unshared error in a dosimetry system, and for generating MCDS in simulations. In our simulations, we also use a slightly extended SUMA model that includes a partially shared component, i.e., errors that are shared among a subset of the person-years. The extended model is

$$X_{i}=\epsilon_{SM}\epsilon_{M,i}\epsilon_{P,p(i)}Z_{i}+\epsilon_{SA}+\epsilon_{A,i},$$

where *p*(*i*) is a partition function that puts person-year *i* into disjoint subsets, and *E*(*ϵ*_*P*,*p*(*i*)_) = 1. In our simulations, we define the partition *p*(*i*) such that *p*(*i*) = *p*(*j*) whenever *i*, *j* are from the same person, so the error component *ϵ*_*P*,*p*(*i*)_ is shared on the individual level. In our simulations, we assume the multiplicative errors are lognormally distributed, and the additive errors are normally distributed.

### Corrected confidence intervals and inference

#### Corrected variance for parameter estimates

In Eq (1), assume we only have access to *Z*_1_ = *E*(*X*_1_), where *X*_1_ = [*X*_1,1_,*X*_1,2_,…,*X*_1,*n*_]^*T*^, *n* is the total number of person-years. Let *θ* = [*a*_0_,*a*_1_,*a*_2_,*a*_3_,*b*_1_,*a*_4_,*a*_5_,*b*_2_]^*T*^. Following the same idea as in Stram et al. [14], the ERR model is fitted using *Z*_1_ instead of *X*_1_. By Fishers Scoring,

$$\hat{\theta }-\theta \approx I_{Z_{1}}^{-1}S_{Z_{1}}.$$

Here, $S_{Z_{1}}$ is the score function, and $I_{Z_{1}}$ is the Fisher information, both computed using *Z*_1_. Since the inverse of the expected information is a constant matrix, we have

$$Var(\hat{\theta })\approx I_{Z_{1}}^{-1}\;Var(S_{Z_{1}})\;I_{Z_{1}}^{-1}.$$

Following the derivation as shown in S1 File, we get

$$\text{Var}(S_{Z_{1}})=I_{Z_{1}}+b_{1}^{2}QG\text{Cov}(X_{1}|Z_{1})GQ^{T}$$

where matrix *Q* = [*Q*_1_,…,*Q*_*n*_] with

$$Q_{i}=\left[\begin{array}{c} 1\\ C_{1,i}\\ C_{2,i}\\ C_{3,i}\\ \frac{Z_{1,i}\exp\left(a_{4}A_{1,i}+a_{5}A_{2,i}\right)}{1+b_{1}Z_{1,i}\exp\left(a_{4}A_{1,i}+a_{5}A_{2,i}\right)+b_{2}X_{2,i}}\\ \frac{b_{1}Z_{1,i}A_{1,i}\exp\left(a_{4}A_{1,i}+a_{5}A_{2,i}\right)}{1+b_{1}Z_{1,i}\exp\left(a_{4}A_{1,i}+a_{5}A_{2,i}\right)+b_{2}X_{2,i}}\\ \frac{b_{1}Z_{1,i}A_{2,i}\exp\left(a_{4}A_{1,i}+a_{5}A_{2,i}\right)}{1+b_{1}Z_{1,i}\exp\left(a_{4}A_{1,i}+a_{5}A_{2,i}\right)+b_{2}X_{2,i}}\\ \frac{X_{2,i}}{1+b_{1}Z_{1,i}\exp\left(a_{4}A_{1,i}+a_{5}A_{2,i}\right)+b_{2}X_{2,i}} \end{array}\right],$$

and *G* = diag([*t*_1_ exp(*a*_0_ + *a*_1_*C*_1,1_ + *a*_2_*C*_2,1_ + *a*_3_*C*_3,1_),…,*t*_*n*_ exp(*a*_0_ + *a*_1_*C*_1,*n*_ + *a*_2_*C*_2,*n*_ + *a*_3_*C*_3,*n*_)]) with *t*_*i*_ being the length of time actually spent in person-year *i*. Let *M* = *QG*, we then have

$$\begin{array}{c} \text{Var}(\hat{\theta })=I_{Z_{1}}^{-1}+b_{1}^{2}I_{Z_{1}}^{-1}M\text{Cov}(X_{1}|Z_{1})M^{T}I_{Z_{1}}^{-1}. \end{array}$$ #(2)

#### Score-type and Wald-type confidence intervals

Using the corrected variance in Eq (2) directly in a score-type inference of $H_{0}:b_{1}=b_{1}^{*}$ leads to the test statistic

$$\begin{array}{c} \frac{{\left({\hat{b}}_{1}-b_{1}^{*}\right)}^{2}}{{\left(I_{Z_{1}}^{-1}+{\left(b_{1}^{*}\right)}^{2}I_{Z_{1}}^{-1}M\text{Cov}(X_{1}|Z_{1})M^{T}I_{Z_{1}}^{-1}\right)}_{b_{1},b_{1}}}. \end{array}$$ #(3)

where we use the subscript *b*_1_,*b*_1_ to index the element of the corrected variance matrix associated with *b*_1_. We term this test as a “score-type” test because the variance of the estimate used in the denominator of the test depends on the null value (specific value of $b_{1}^{*}$ in *H*_0_) being tested. Note however that *M* and $I_{Z_{1}}^{-1}$ are evaluated at $\hat{\theta }$.

Note also that as $b_{1}^{*}$ approaches ±∞, this statistic asymptotes to $1/{\left(I_{Z_{1}}^{-1}M\text{Cov}(X_{1}|Z_{1})M^{T}I_{Z_{1}}^{-1}\right)}_{b_{1},b_{1}}$, which can sometimes fall below the critical value. Under these situations, the score-type test-based confidence intervals will include both positive and negative infinity, which is undesirable for dose effect assessment.

Stram et al. [14] used the corrected variance to calculate a 95% Wald-type confidence interval which, when applied to our models, translates to

$$\begin{array}{c} {\hat{b}}_{1}\pm z_{\frac{\alpha }{2}}\;\sqrt{{\left(I_{Z_{1}}^{-1}+{\left({\hat{b}}_{1}\right)}^{2}I_{Z_{1}}^{-1}M\text{Cov}(X_{1}|Z_{1})M^{T}I_{Z_{1}}^{-1}\right)}_{b_{1},b_{1}}}. \end{array}$$ #(4)

This is termed a Wald-type confidence interval because the variance term used is a constant once the parameters have been estimated, and does not depend on the null value $b_{1}^{*}$ in *H*_0_. While this Wald-type confidence interval does not have the extreme-value problem, it ignores the fact that the variance of the estimator is greatly affected by the effect size *b*_1_, which can lead to inadequate and unbalanced confidence interval coverage.

#### Inference distributions for parameter estimates

Our proposed method is based on the results of a simple theorem stated below.

**Theorem 1** Consider the SUMA model *X*_*i*_ = *Z*_*i*_*ϵ*_*SM*_*ϵ*_*M*,*i*_ + *ϵ*_*SA*_ + *ϵ*_*A*,*i*_, 1 ≤ *i* ≤ *n*, where all *ϵ*’s are independent with $\text{E}(\epsilon_{SM})=\text{E}(\epsilon_{Mi})=1,\;\text{E}(\epsilon_{SA})=\text{E}(\epsilon_{Ai})=0,\;\text{Var}(\epsilon_{Mi})=\sigma_{M}^{2},\;\text{Var}(\epsilon_{Ai})=\sigma_{A}^{2}$, *X*_*i*_ is the unobserved true dose, *Z*_*i*_ = E(*X*_*i*_) is known, and *n* is the number of subjects, and the simple linear regression model *Y*_*i*_ = *a* + *bX*_*i*_ + *ϵ*_*i*_, where *ϵ*_*i*_’s are independent with E(*ϵ*_*i*_) = 0, Var(*ϵ*_*i*_) = *σ*^2^, and *ϵ*_*Mi*_,*ϵ*_*Ai*_,*ϵ*_*i*_ are mutually independent. The MLE slope estimator ${\hat{b}}_{n}$ with the model fitted using *Z*_*i*_’s has the following properties

1. When $\sigma_{M}^{2}=0,\text{E}(Z_{i}^{2})<\infty$ and $\max_{1\leq i\leq n}Z_{i}{=}_{p}o\left(n^{\frac{1}{2}}\right)$, we have
  $${\hat{b}}_{n}\to_{d}b\epsilon_{SM}+N,$$
  where *N* is a random variable with distribution
  $$N∼\text{Gaussian}\left(0,\frac{\sigma^{2}+\sigma_{A}^{2}}{n\;\text{Var}(Z)}\right),\;N⊥\epsilon_{SM}.$$
2. When $\sigma_{M}^{2}>0,E(Z_{i}^{4})<\infty$ and $\max_{1\leq i\leq n}Z_{i}^{2}{=}_{p}o\left(n^{\frac{1}{2}}\right)$, we have
  $${\hat{b}}_{n}\to_{d}b\epsilon_{SM}N^{\prime }+N,$$
  where *N*′,*N* are random variables with distributions
  $$N^{\prime }∼\text{Gaussian}\left(1,\frac{\text{E}(Z^{2}{\left(Z-\text{E}Z\right)}^{2})}{n\;\text{Var}{\left(Z\right)}^{2}}\sigma_{M}^{2}\right),\;N∼\text{Gaussian}\left(0,\frac{\sigma^{2}+\sigma_{A}^{2}}{n\;\text{Var}(Z)}\right),$$
  respectively, and *ϵ*_*SM*_,*N*′,*N* are independent.

*Proof*. See supplementary material provided (S1 File).

The theorem says that the distribution of the slope estimator under a linear model is greatly influenced by the distribution of the shared error *ϵ*_*SM*_. When all multiplicative errors are strictly positive, it can be seen from the proof that we can replace *N*′ with a truncated normal random variable that is strictly positive. In such case, we can easily show that the 1-*α* confidence intervals are always bounded, noting that the probability of *bϵ*_*SM*_*N*′ + *N* being less than $\hat{b}$ approaches 0 as *b* increases. We note that the theorem, although proved only for the simple linear regression model, naturally extends to a general linear dose effect model, such as the model in Eq (1).

Dealing with the exact distribution of $\hat{b}$ is difficult since it involves the multiplication of two random variables and a summation, and not necessary since a real MCDS will deviate from the ideal SUMA model which this exact distribution is based on. For practical purposes, we suggest approximating the distribution of $\hat{b}$ as *bL*+*N* where *L* is a lognormal random variable with E(*L*) = 1, and *N* a normal random variable with E(*N*) = 0. A lognormal distribution is very natural for the shared multiplicative error. In a complex dosimetry system, there can be many different shared (almost always multiplicative) error components, so that on the log scale, from the central limit theorem, an approximate normal distribution can be assumed for an overall combined shared error, as represented by *ϵ*_*SM*_.

We use this *bL*+*N* mixture distribution as the inference distribution of ${\hat{b}}_{1}$ in Eq (1). It follows that $\text{Var}({\hat{b}}_{1})=b_{1}^{2}\text{Var}(L)+\text{Var}(N)$. Comparing it with Eq (2), we let

$$\begin{array}{c} \text{Var}(L)={\left(I_{Z_{1}}^{-1}M\;\text{Cov}(X_{1}|Z_{1})M^{T}I_{Z_{1}}^{-1}\right)}_{b_{1},b_{1}},\;\text{Var}(N)={\left(I_{Z_{1}}^{-1}\right)}_{b_{1},b_{1}}. \end{array}$$ #(5)

For the other parameters, denoted as *a*, including *a*_0_ to *a*_5_ and *b*_2_, that are associated with known covariates, we base our inference on a normal distribution rather than the mixture one. The rationale is, when there is only shared multiplicative error on dose, measured dose is just a rescaled version of true dose. Thus, the likelihood function would be the same as if there is no error on dose if we rescale the parameter associated with dose while leaving all other parameters unchanged. This suggests shared multiplicative error will have little influence on these parameters. Meanwhile, the theorem above indicates that unshared error introduces random normal components to the estimator. Thus, we propose to use a standard normal approximation, $\hat{a}=a+N$ for inference about all parameters except the dose effect. For these parameters, the variance of *N* still needs to be corrected and is different from what we get from inverse information matrix when *b*_1_ > 0. From Eq (2), we let

$$\begin{array}{c} \text{Var}(N)={\left(I_{Z_{1}}^{-1}\right)}_{a,a}+\;{\hat{b}}_{1}^{2}{\left(I_{Z_{1}}^{-1}M\;\text{Cov}(X_{1}|Z_{1})M^{T}I_{Z_{1}}^{-1}\right)}_{a,a}. \end{array}$$ #(6)

In the following, we refer to our proposed inference method shown in Eqs (5) and (6) as *bL*+*N*, though for parameters other than *b*_1_, we are only using a normal distribution.

#### Corrected confidence intervals construction

Using the inference distributions determined above, for the dose effect *b*_1_, we can calculate the probability of obtaining an estimate equal to or more extreme than the observed value ${\hat{b}}_{1}$ under $H_{0}:b_{1}=b_{1}^{*}$, which is $2\times \min\;(P(b_{1}^{*}L+N\leq {\hat{b}}_{1}),\;P(b_{1}^{*}L+N\geq {\hat{b}}_{1}))$. It follows that the lower and upper confidence limits *b*_1,*l*_,*b*_1,*u*_ can be found for a two-sided *α*-level confidence intervals, satisfying

$$\text{P}(b_{1,l}L+N\geq {\hat{b}}_{1})=\alpha /2,\;\text{P}(b_{1,u}L+N\leq {\hat{b}}_{1})=\alpha /2.$$

For other parameter estimates, again denoted as *a*, the confidence limits are calculated as

$$\hat{a}\pm z_{\alpha /2\;}\;\sqrt{{\left(I_{Z_{1}}^{-1}\right)}_{a,a}+\;{\hat{b}}_{1}^{2}{\left(I_{Z_{1}}^{-1}M\;\text{Cov}(X_{1}|Z_{1})M^{T}I_{Z_{1}}^{-1}\right)}_{a,a}}$$

#### Efficient calculation of the corrected variance

In the Mayak Worker Cohort, there are as many as 25,575 individuals with up to 60 years of follow-up to date. The full-cohort analyses described below are based on person-year data with roughly 350,000 individual person-year dose contributions. In this case Cov(*X*_1_|*Z*_1_) has more than 60 billion distinct values and direct computation of or even storing this matrix in computer memory can be challenging. However, to compute the corrected variance of the parameter estimates, we do not need to calculate Cov(*X*_1_|*Z*_1_) directly. Let *X*_1_ be a matrix with each column a single dose replication of *X*_1_. Assume *X*_1_ is of dimension *n* × *k*, and *M* is of dimension *p* × *n*. The *p* × *p* matrix of primary interest *M*Cov(*X*_1_|*Z*_1_)*M*^*T*^ can be estimated as the sample covariance matrix of the more manageable intermediate *n* × *k* matrix *W* = *M X*_1_. To see this, observe that

$$\begin{array}{l} \text{Cov}(W)=\frac{1}{n-1}(W^{T}W-\frac{1}{n}W^{T}11^{T}W)\\ \;=\frac{1}{n-1}(MX_{1}X_{1}^{T}M^{T}-\frac{1}{n}MX_{1}11^{T}X_{1}^{T}M^{T})\\ \;=M\frac{1}{n-1}(X_{1}X_{1}^{T}-\frac{1}{n}X_{1}11^{T}X_{1})M^{T}\\ \;=M\;\hat{\text{Cov}}(X_{1}|Z_{1})M^{T} \end{array}$$

The time complexity of calculating this product is reduced to Θ(*pnk* + *p*^2^*k*) from Θ(*n*^2^*k* + *p*^2^*n*^2^), which is a significant improvement when *n* is dominantly large.

#### Inference for the null model

Under the null hypotheses *H*_0_: *b*_1_ = 0, the dose modifying effects of age and sex, i.e. *a*_4_, *a*_5_ are no longer identifiable. To be able to make proper tests about *H*_0_, we perform the score test with *a*_4_ = *a*_5_ = 0 fixed in Eq (1), i.e. a reduced model below,

$$\begin{array}{c} h_{i}=\exp\left(a_{0}+a_{1}C_{1,i}+a_{2}C_{2.i}+a_{3}C_{3,i}\right)(1+b_{1}X_{1,i}+b_{2}X_{2,i}). \end{array}$$ #(7)

We choose the score test over the Wald test because the latter requires convergence of the model fitting procedure, which is often not possible under *H*_0_.

### Simulations

We use Eq (1) as the ERR model for all our simulations. This model is a simplification of models used in analyses of lung cancer risks in the MWC [26].

#### Dose simulation

For the simulation studies, five dosimetry systems are considered. These include four SUMA dosimetry systems with only multiplicative errors. For each of the SUMA dosimetry systems, we used the average cumulative annual internal dose from MWDS-2013 as the mean dose *Z*_1_. The SUMA dosimetry systems used in the simulations have error components as listed in Table 2, where $\sigma_{SM}^{2}=\text{Var}(\epsilon_{SM}),\sigma_{M}^{2}=\text{Var}(\epsilon_{M,i}),\sigma_{P}^{2}=\text{Var}(\epsilon_{P,p(i)})$.

**Table 2:** Simulation settings for the SUMA dosimetry system.

Dosimetry system	True dose (*X*_1,*i*_)	$\sigma_{SM}^{2}$	$\sigma_{M}^{2}$	$\sigma_{P}^{2}$
DS-S	*ϵ*_*SM*_*Z*_1,*i*_	0.318	0	0
DS-U	*ϵ*_*M*,*i*_*Z*_1,*i*_	0	0.223	0
DS-SU	*ϵ*_*SM*_*ϵ*_*M*,*i*_*Z*_1,*i*_	0.318	0.223	0
DS-SUP	*ϵ*_*SM*_*ϵ*_*M*,*i*_*ϵ*_*P*,*p*(*i*)_*Z*_1,*i*_	0.318	0.223	0.200

For each of the SUMA dosimetry systems, we generated 1,000 dose replications. We also used the actual MWDS-2013, in which case a total of 1,000 dose replications were generated by the dosimetrists and provided to us. As in Kwon et al. [15], for each simulation experiment, one of the realizations was used to generate the outcome survival data. The remaining 999 replications were used to obtain the mean doses used in model fitting, and to calculate *M*Cov(*X*_1_|*Z*_1_)*M*^*T*^ used for the adjusted confidence intervals. The simulation is repeated for each dose replication, chosen as the true dose for generating the outcome. The particular values of the variance components given in the table were chosen as similar in value to shared and unshared error estimates for the Hanford Thyroid Disease Study CIDER dosimetry obtained by Stram and Kopecky [27].

For external dose, we used the average cumulative annual external dose in MWDS-2013 as the true dose for all simulations. For the other covariates, i.e. sex and age, we used the data from MWC directly.

#### Survival data simulation

For each participant in the MWC, we generated an event time following a piecewise exponential distribution specified by Eq (1). Here the assumed hazard function is constant throughout each person-year. This event time was compared to the actual end of follow-up for this MWC member. If the generated event time was less than the end of follow-up, the participant was treated as a case at the generated failure time, otherwise the participant was right-censored at their end of follow-up. Since last follow-up time is pre-defined from the MWC data, this is non-informative censoring and will not introduce bias to our analysis. To use Poisson regression for this survival data, we tabulated the generated follow-up time, event indicator, and covariate information for each person-year of follow-up (approximately 125,000 total in any given simulation).

We considered three models defined in terms of the baseline rate (*a*_0_) and internal dose effect (*b*_1_) for men at age 60 as described in Table 3. For the moderate and strong models, we used all 5 dosimetry systems. For the null model, we used MWDS-2013 only. The other parameters in the model were taken as in Table 4. Here sex is coded with female being 1, and male being 0. We evaluate the moderate and strong models for each of the five dosimetry systems described above.

**Table 3:** Simulation settings for *a*_0_ and *b*_1_.

Model	Baseline rate (100,000×exp(*a*_0_)) (cases per 100,000 person years)	*b*_1_/Gy	Expected cases	Expected dose-associated cases
Null	100,000×exp(-5.0) = 673.8	0	≈800	0
Moderate	100,000×exp(-6.5) = 150.3	3.6	≈300	≈100
Strong	100,000×exp(-5.0) = 673.8	3.6	≈1,200	≈400

**Table 4:** Simulation settings for parameters other than *a*_0_ and *b*_1_.

	Baseline rates			Internal dose effect modifiers		External Dose
Risk factor	log(age/60)	log^2^(age/60)	sex	log(age/60)	sex	
Parameter	*a*_1_	*a*_2_	*a*_3_	*a*_4_	*a*_5_	*b*_*2*_ /Gy
Value	5.64	-6.39	log(0.5)	-3.15	1.33	0.21

#### Estimation and inference

With *Z*_1_, the average dose of the 999 dose replications that are not used for generating the outcome, we used Fisher’s scoring to obtain parameter estimates $\hat{\theta }$ and the information $I_{Z_{1}}$. We calculated 4 types of confidence intervals (CI) for the dose response parameter of interest *b*_1_. Naïve CI’s are the usual Wald CI’s using the variance estimated without correction for measurement error. The other three CI’s all use the corrected variance given in Eq (2). As described above, Eq (4) was used for Wald-type CI’s, Eq (3) was used for score-type CI’s, and Eq (5) was used for the *bL*+*N* CI’s. For parameters other than *b*_1_, we used only the Wald-type confidence intervals.

In Eq (2), Matrix *M* was calculated based on $\hat{\theta }$. We used the same 999 dose replications to calculate *M*Cov(*X*_1_|*Z*_1_)*M*^*T*^. Confidence intervals are constructed as described previously. Overall coverage is calculated as the percentage of the confidence intervals covering the true value in all 1,000 simulations with each dosimetry system. In describing the results, we focused on the coverage of 95% confidence intervals.

We also used the true dose *X*_1_ in the above simulations for model fitting, hypothesis testing, and confidence interval construction, for comparison purposes.

## Results

### Moderate and strong ERR model

#### Coverage of confidence intervals

Overall coverage of the various 95% CI’s for *b*_1_ for both the moderate and strong models and the 5 different dosimetry systems is given in Table 5. When there is only unshared error (DS-U), in the moderate model, there is almost no difference in coverage between the naïve CI and the corrected CI’s, all being around 0.95. In the strong model, the coverage for all CI’s is decreased to around 0.91 and there are only very small differences among them. In the presence of shared error (DS-S, DS-SU, DS-SUP), the coverage of the naïve CI is poor, ranging from 0.436 to 0.703 for the two models, compared to the desired value of 0.95. The coverage of all the corrected CI’s is much better, while there is a noticeable difference in performance among the three correction methods. The coverage of the Wald-type CI’s is around 0.88, and the CI’s are biased towards the null; the lower limits are below the true values in all simulations, and the upper limits are below the true value in around 11% of all simulations. The score-type CI’s have better coverage. However, the coverage is around 0.97, slightly higher than the desired value of 0.95. The upper limits of the score-type CI’s are almost always above the true values in all simulations. We also notice there is one simulation using the moderate model with DS-SUP, where the asymptote of the test statistic is below the critical value, giving an unbounded CI. The coverage of the *bL*+*N* confidence intervals are very close to 0.95. However, the coverage is slightly asymmetric, especially for DS-SUP; when the confidence interval fails to include the true value, it is more likely to be above the upper limit than below the lower limit.

**Table 5:** Confidence interval coverage of different methods for *b*_1_ in moderate and strong model.

Dosimetry system	ERR model	Confidence intervals			
		Corrected			Naïve
		Wald-type	Score-type	*bL*+*N*	
DS-U	Moderate	.947 (.053, .000)†	.949 (.050, .001)	.949 (.050, .001)	.945 (.054, .001)
	Strong	.910 (.087, .003)	.918 (.079, .003)	.918 (.079, .003)	.906 (.091, .003)
DS-S	Moderate	.860 (.140, .000)	.975 (.002, .023)	.943 (.039, .018)	.663 (.254, .083)
	Strong	.898 (.102, .000)	.960 (.000, .040)	.947 (.027, .026)	.436 (.358, .206)
DS-SU	Moderate	.885 (.115, .000)	.972 (.001, .027)	.941 (.040, .019)	.677 (.251, .072)
	Strong	.882 (.118, .000)	.973 (.000, .027)	.957 (.025, .018)	.448 (.401, .151)
DS-SUP	Moderate‡	.892 (.108, .000)	.985 (.000, .015)	.956 (.034, .010)	.703 (.230, .067)
	Strong	.870 (.130, .000)	.982 (.000, .018)	.951 (.037, .012)	.467 (.383, .150)
MWDS-2013	Moderate	.894 (.106, .000)	.964 (.029, .007)	.933 (.061, .006)	.830 (.151, .019)
	Strong	.894 (.106, .000)	.960 (.032, .008)	.936 (.060, .004)	.624 (.300, .076)

The coverage of confidence intervals for internal dose effect *b*_1_ using different methods in moderate and strong ERR models is given, with 5 dosimetry systems.

† Overall coverage (fraction of times the upper bound is below the true value, fraction of times the lower bound is greater than the true value).

‡ Simulation #662 was excluded because score-type confidence interval included ±∞.

In all 4 SUMA dosimetry systems (DS-U, DS-S, DS-SU, DS-SUP), the correction has virtually no effect on CI coverage for parameters other than *b*_*1*_ (S1 Table), and nearly all are close to 0.95. Coverage of naïve CI’s using the true dose *X*_1_ instead of *Z*_1_ for model fitting are given in the supplementary materials (S2 Table).

With MWDS-2013 (Table 5), the coverage of naïve CI is poor, being only 0.624 for the strong model and 0.830 for the moderate model. The Wald-type CI has coverage 0.894. Again, the Wald CI is biased towards the null, with the same pattern as seen using the SUMA dosimetry systems. The coverage of score-type CI’s is around 0.96 and the coverage of *bL*+*N* CI’s is around 0.93. Both the score-type method and the *bL*+*N* method have asymmetric CI’s; the lower limits of both methods are a little conservative while *bL*+*N* has slightly inadequate upper limits.

Overall coverage of the 95% CI’s for the parameters other than *b*_1_ when using the MWDS-2013 dosimetry system is given in Table 6. The coverage of corrected CI’s for these parameters is around 0.95 while naïve CI’s have inadequate coverage for *b*_2_ in both the moderate and strong models and for almost all parameters in the strong model.

**Table 6:** Confidence interval coverage for model parameters with MWDS-2013 in moderate and strong models.

Parameter	Naïve Confidence Interval		Corrected Confidence Interval	
	Moderate	Strong	Moderate	Strong
*a*_0_	.943 (.015, .042)†	.906 (.028, .066)	.956 (.011, .033)	.946 (.017, .037)
*a*_1_	.954 (.032, .014)	.924 (.050, .026)	.956 (.031, .013)	.937 (.044, .019)
*a*_2_	.966 (.014, .020)	.961 (.020, .019)	.967 (.013, .020)	.965 (.017, .018)
*a*_3_	.936 (.019, .045)	.917 (.016, .067)	.944 (.012, .044)	.941 (.010, .049)
*a*_4_	.946 (.034, .020)	.916 (.061, .023)	.959 (.024, .017)	.958 (.033, .009)
*a*_5_	.945 (.040, .015)	.914 (.072, .014)	.957 (.033, .010)	.941 (.050, .009)
*b*_2_	.914 (.076, .010)	.876 (.079, .045)	.942 (.054, .004)	.964 (.021, .015)

The coverage of confidence intervals (CI) for model parameters other than dose effect *b*_1_ are given. Naïve CI and *bL*+*N* CI were used for both moderate and strong ERR models with MWDS-2013.

† Overall coverage (fraction of times the upper bound is below the true value, fraction of times the lower bound is greater than the true value).

#### Bias of ${\hat{b}}_{1}$

Means and standard deviations of ${\hat{b}}_{1}$ are given in Table 7 for the moderate and strong models. For the strong model, there is a statistically significant bias towards the null in the slope estimates except when there are only shared errors (i.e. model DS-S). With the SUMA dosimetry systems, we also see that the standard deviation of ${\hat{b}}_{1}$ is much larger when shared errors are present (DS-S, DS-SU, DS-SUP), reflecting the effect of shared error as described in Theorem 1.

**Table 7:** Mean, standard deviation and *p*-value for ${\hat{b}}_{1}$.

Dosimetry System	Moderate		Strong	
	*b*_1_	*p*	*b*_1_	*p*
DS-U	3.65 (0.97)	0.094	3.43 (0.50)	<10^−4^
DS-S	3.77 (2.19)	0.013	3.58 (1.77)	0.717
DS-SU	3.62 (1.98)	0.802	3.39 (1.66)	<10^−4^
DS-SUP	3.43 (1.89)	0.004	3.11 (1.4)	<10^−4^
MWDS-2013	3.61 (1.48)	0.813	3.33 (0.99)	<10^−4^

Means of *b*_1_ estimates are given for moderate and strong model in 5 dosimetry systems. Standard deviations of the estimates and the p-values of the t-test of *H*_0_: *b*_1_ = 3.6 (true value) are given.

### The null model

Under the null ERR model *b*_1_ = 0, the performance of the score test is given in Table 8. There is very little effect of measurement error on the performance of the score test. The score test is a little unbalanced, with less than desired rejections on the negative end, especially in the moderate model. This unbalance is seen in the score test using either *Z*_1_ or *X*_1_.

**Table 8:** Score test of *H*_0_: *b*_1_ = 0 for the null model with MWDS-2013.

Internal dose‡	Moderate Model	Strong Model
*Z*_1_ (with error)	.967 (.008, .025)†	.954 (.021, .025)
*X*_1_ (without error)	.970 (.006, .024)	.958 (.013, .029)

Result of score test for the null ERR model is given. The score test was performed on the reduced model, given in Eq (7).

† Fraction of times *H*_0_ is not rejected (fraction of times the test statistic is significantly negative, fraction of times the test statistic is significantly positive).

‡ Interval dose used for model fitting, score and information calculation under *H*_0_: *b*_1_ = 0.

## Discussion

We evaluated the performance of the various confidence intervals (Wald-type, score-type, *bL*+*N* and naïve) in simulation experiments using several dosimetry systems, including the actual Mayak Workers Dosimetry System 2013 (MWDS-2013), with results given in Table 5 and Table 6. Overall, using our proposed methods, we saw marked improvement in the coverage of 95 percent CI’s for the dose-response parameter (*b*_1_) associated with the variable *X*_*1*_, that is affected by measurement error. For example, with the internal dose replications from MWDS-2013 in the strong model (about 1,200 cancer cases, 400 of which were due to exposure), the 95% *bL*+*N* corrected CI’s included the true value of the dose response parameter 93.6 percent of the time, in contrast to just 62.4 percent of the uncorrected CI’s. In experiments with simplifications of this system (the various SUMA dosimetry systems) we saw even larger improvements. Compared to our *bL*+*N* method, the corrected Wald-type CI’s performed worse in all our simulations, improving coverage only to around 0.89. This clearly is due to ignoring the dependence of the corrected variance on the parameter being estimated (*b*_*1*_), leading to conservative lower limits and inadequate (overly liberal) upper limits. In the SUMA dosimetry systems, the upper limits of score-type CI’s are overly conservative, rarely going below the true value. This suggests that the normal distribution, which score-type CI’s are based on, does not model the distribution of the estimator properly in these situations. However, this is not reflected when MWDS-2013 is used, where score-type CI’s have somewhat better upper limits compared to *bL*+*N*. A typical comparison between the score-type CI and the *bL*+*N* CI in one of the simulations using the moderate model with MWDS-2013 is shown in Fig 1. Overall, compared to score-type CI’s, the proposed *bL*+*N* CI’s have superior performance when used with simplified dosimetry systems, and provide very comparable result when MWDS-2013 is used, while eliminating the possibility of finding CI’s that include ±∞.

**Fig 1:**
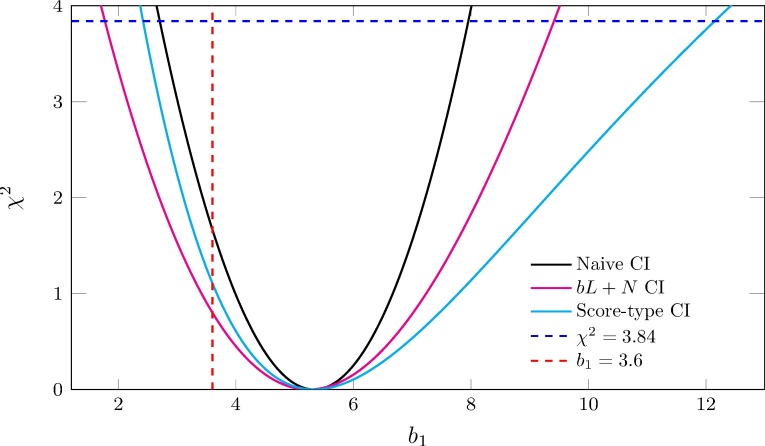
Comparison of naïve CI, score-type CI and *bL*+*N* CI. Confidence intervals (CI’s) in one simulation with the moderate model with MWDS-2013 are shown. In the plot, the *p*-values of *b*_1_ at different points are evaluated using the distributions underlying each method and transformed into *χ*^2^ test statistics.

We note, for *bL*+*N* CI’s, even though overall coverage was excellent, the CI’s for the risk parameter *b*_1_ in the ERR model were unbalanced; they tended to be conservative on the low end, and were overly liberal on the high end. This may be due to the dependence of the information matrix and matrix *M* on the parameter estimates, an aspect we ignored in our variance calculation, as both are only evaluated at $\hat{\theta }$. We observed that, with or without dosimetry error, the standard error of the dose response parameter *b*_1_ in an ERR model appears to increase with *b*_1_. An example is given in Fig 2 from one simulation where we see essentially a linear increase in the uncorrected variance of ${\hat{b}}_{1}$. Interestingly the correction term ${\left(I_{Z_{1}}^{-1}M\text{Cov}(X_{1}|Z_{1})M^{T}I_{Z_{1}}^{-1}\right)}_{b_{1},b_{1}}$ decreases with *b*_1_, countering somewhat the increase in total variance of this parameter’s estimate ($b_{1}^{2}$ multiplied by the correction term). S2 Table shows that, even when there are no dose errors, the standard Wald test confidence intervals for the two dose response parameters, *b*_1_ and *b*_2_, have unbalanced coverage similar in pattern to what is seen in Table 5 and Table 6. We therefore attribute at least part of the imbalance in our corrected confidence intervals to be due to the structure of the risk model, rather than the measurement errors. Most likely this is due to ignoring the dependence of the information matrix on the parameters being estimated. A full treatment of this issue is deferred for later work since we note that treating the information matrix and the matrix *M* as a function of *b*_1_ would add considerable computational burden to our approach.

**Fig 2:**
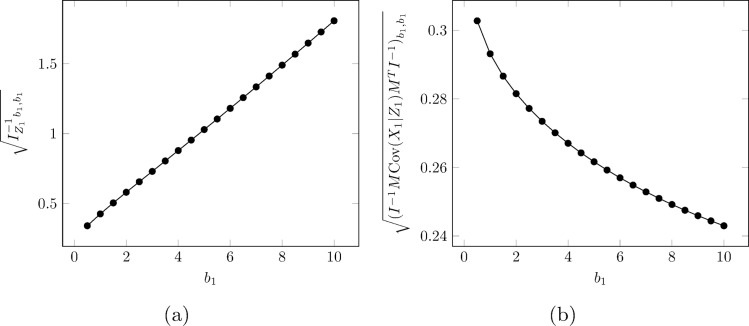
Variance components of *b*_1_ vs. *b*_1_. Subfigure (a) shows the change of $\sqrt{I_{b_{1},b_{1}}^{-1}},\;$ the naïve standard error of *b*_1_, with respect to *b*_1_. Subfigure (b) shows the change of $\sqrt{{\left(I_{Z_{1}}^{-1}M\text{Cov}(X_{1}|Z_{1})M^{T}I_{Z_{1}}^{-1}\right)}_{b_{1},b_{1}}}$ with respect to *b*_1_. In our current method, both are fixed, with values calculated at $\hat{\theta }$.

We noticed that the *bL*+*N* confidence intervals clearly outperformed the score-type confidence intervals (especially on the upper side) whenever there was shared error in the SUMA dosimetry systems (lines in Table 5 corresponding to DS-S, DS-SU, DS-SUP) however this was not seen for the MWDS-2013 system. This may partly be because the variance of the shared errors in MWDS-2013 are not as large as those simulated in the SUMA dosimetry systems (we infer this because the naïve CI’s performed better under the MWDS-2013). In addition, the underlying assumption of the *bL*+*N* approximation is that the shared errors are lognormally distributed. This holds exactly for the SUMA systems used, but may not necessarily apply to the MWDS-2013. Indeed, some preliminary work not yet shown seems to indicate that the shared component of error in this dosimetry system may be less skewed than would be a lognormal with the same mean and variance. Additional work on incorporating a more flexible model for the distribution of the shared error components that can be empirically tuned to the realizations from a given system, may be warranted.

The biases in parameter estimates that we saw for the strong model in Table 7 is likely due to the dilution effect, described by Prentice [28] in his discussion of survival analysis in the presence of random covariate errors. The attenuation in the risk estimate occurs because highly exposed individuals are more likely to have an early event, implying that the distribution of true dose given estimated dose has a lower mean value in the later time periods than in the earlier time periods. Since the dosimetry system does not use information from the response this results in a biased estimate of the slope parameter ${\hat{b}}_{1}$. In the strong model about 5 percent of all individuals (∼400/7873) die because of exposure, whereas in the moderate model this is reduced to about 1.25 percent, and the dilution bias diminishes accordingly. The dilution effect is not dependent upon the size of the shared errors. Rather, it reflects random unshared errors; the dosimetry system with the greatest dilution (DS-SUP) has the most random error and the one with only shared error (DS-S) does not show a dilution effect. The latter follows because for DS-S everyone at a given estimated dose level has the same (unknown) true dose, so that the distribution of true dose given estimated dose is not dependent on time. In many studies exposure-associated cases arise in only a very small fraction of the total number of individuals considered. For example, in the A-bomb study the excess number of radiation-associated solid tumor cases over 40 years of follow-up (1958–1998) was estimated to be only about 2 percent of the exposed members of the cohort (http://www.rerf.jp/radefx/late_e/cancrisk.html). Our moderate model, which does not show significant dilution effects, is designed to give an excess number of cases which is very close to the actual Mayak analysis (about 100 cases).

When *b*_1_ = 0, the corrected variance is the same as the uncorrected variance, so that the naïve score test is still valid once the dose modifier variables are dropped from the model since they are not identifiable if *b*_1_ = 0. We only considered the score test since the other tests depend on model convergence which is not obtainable in a large fraction of the simulations when no dose effect exists.

Our approach uses mean dose from the dosimetry system to fit the ERR model and then corrects the confidence limits of the parameters. That is, we do not use the Monte Carlo realizations themselves to fit the model, only to correct the confidence limits of the fitted parameters. Other methods have been proposed for the problem of shared dosimetry error in epidemiological studies [8, 15]. Kwon et al. [15] fit the risk model to each realization in turn and then resample realizations with weights dependent on the likelihood of each fit. Contrary to our results, they found that use of the mean doses from the dosimetry system provided very poor estimates of the risk parameter, even for nearly linear dose response (they used a model with linear dose response on the odds scale). In some of our simulations the risk estimates were biased towards the null, due most likely to the dilution effect. The bias is modest or absent for our more realistic moderate model. Even for the strong model the biases in the risk estimate were not great enough to disturb the overall coverage of the corrected CI’s. The simulations conducted by Kwon et al. did not simulate survival times, rather only a binary event, so that the dilution cannot explain their findings; we do not yet understand why they should have seen this effect.

## Conclusions

We proposed using a mixture distribution of lognormal and normal components, referred to as *bL*+*N*, to approximate the distribution of dose effect estimates in a generalized ERR model, which can be used for the construction of hypothesis tests and confidence intervals. These CI’s are improved over Wald-type CI’s since they (appropriately) allow the variance of the parameter estimates to change with dose effect *b*_1_. Moreover, these mixture-corrected CI’s, unlike score-type corrected CI’s, will never include ±∞.

To date all the studies we are familiar with, that use Monte-Carlo dosimetry systems, are in radiation epidemiology. We believe, however, that the proposed method as described and implemented here will have important applications in other areas of epidemiological study that may benefit from the use of Monte Carlo dosimetry systems. Dose calculation for air pollution studies, to give one example, is an additional area where shared errors are a prominent feature of the dosimetry system. This paper can be used as guidance for incorporating multiple realizations into dose response estimation and inference in a modeling framework that allows for considerable flexibility in dose response estimation.

An R package has been implemented to facilitate further research and applications (https://github.com/zhuozhang/Rerr). For future work, we will consider allowing ERR models with more than one error prone covariate such as the external dose in MWDS-2013. This would, as a special case, allow for error correction of confidence intervals for linear quadratic dose response ERR models, which are also commonly used in radiation epidemiology.

## Supporting information

S1 File Derivations and proofs. This file shows 1) the equivalence of survival analysis with piecewise exponential hazard function and tabulated Poisson regression, 2) derivation of Eq (2), and 3) proof of Theorem 1.
(DOCX)

S1 Table Confidence interval coverage for all model parameters with SUMA dosimetry systems. The coverage of confidence intervals for all parameters in Eq (1) using different methods in moderate and strong ERR models is given, with DS-U, DS-S, DS-SU, DS-SUP.
(DOCX)

S2 Table Confidence interval coverage for model parameters fitted with true dose. The coverage of confidence intervals for all parameters in Eq (1) is given. The model was fitted using the true internal dose *X*_1_ in both moderate and strong ERR models with 5 dosimetry systems.
(DOCX)
