# Supplementary material for: Correction of confidence intervals in excess relative risk models using Monte Carlo dosimetry systems with shared errors
Source: PLoS One. 2017 Apr 3;12(4):e0174641. doi: 10.1371/journal.pone.0174641 (PMC5378348; doi:10.1371/journal.pone.0174641)
Supplement: S1 File — This file shows 1) the equivalence of survival analysis with piecewise exponential hazard function and tabulated Poisson regression, 2) derivation of Eq (2), and 3) proof of Theorem 1. (DOCX) [file pone.0174641.s001.docx]

This file shows 1) the equivalence of survival analysis with piecewise exponential hazard function and tabulated Poisson regression, 2) derivation of Equation (2), and 3) proof of Theorem 1.

# Equivalence of survival analysis with piecewise exponential hazard function and tabulated Poisson regression

We rewrite the model defined in Equation (1) as below

$$h_{i,j}=\exp\left( a_{0}+a_{1}C_{1,i,j}+a_{2}C_{2.i,j}+a_{3}C_{3,i,j} \right)\left( 1+b_{1}X_{1,i,j}\exp{(a}_{4}A_{1,i,j}+a_{5}A_{2,i,j})+b_{2}X_{2,i,j} \right)$$

where *i* indexes individual, *j* indexes time interval. For individual *i*, let the disease status be *D_i_* at the end of follow-up with follow-up consisting of time interval $t_{i,j}$’s with *j* ranging from 1 to *J_i_*. The likelihood for survival analysis is

$$L\left( \theta\right)=\prod_{i=1}^{n} \exp\left( -\sum_{j=1}^{J_{i}} h_{i,j}t_{i,j} \right)h_{i,J_{i}}^{D_{i}}$$

Now for individual *i* we construct independent Poisson random variables $D_{i,j}\sim\text{Poisson}\left( h_{i,j}t_{i,j} \right)$ with the same *i*,*j* definition as above. $D_{i,j}$ is assigned 0 for $j=1,\ldots,J_{i}-1$, and *D_i_* for $j=J_{i}$. For these Poisson random variables, the likelihood contributed by individual $i$ will be

$$L_{i}\left( \theta\right)=\exp\left( -h_{i,J_{i}}t_{i,J_{i}} \right)\left( h_{i,J_{i}}t_{i,J_{i}} \right)^{D_{i}}\prod_{j=1}^{J_{i}-1} \exp\left( -h_{i,j}t_{i,j} \right)=\exp\left( -\sum_{j=1}^{J_{i}} h_{i,j}t_{i,j} \right)h_{i,J_{i}}^{D_{i}}t_{i,J_{i}}^{D_{i}}$$

The full likelihood function for the constructed Poisson data and the likelihood for the survival analysis only differ by a constant factor of $\prod_{i=1}^{n} t_{i,J_{i}}^{D_{i}}$.

# Derivation of Equation (2)

For Equation (1), we have

$$S_{Z_{1}}=Q\left( D-\text{E}_{Z_{1}}\left( D \right) \right),$$

$$I_{Z_{1}}=Q{\left[ \begin{matrix} \text{E}_{Z_{1}}(D_{1}) & & \\ & \ddots& \\ & & \text{E}_{Z_{1}}\left( D_{n} \right) \end{matrix} \right]Q}^{T}=Q \text{diag}\left( \text{E}_{Z_{1}}(D) \right)Q^{T},$$

where *D* is a vector of tabulated outcome data,

$$D=\left[ \begin{matrix} D_{1} \\ \vdots\\ D_{n} \end{matrix} \right],$$

and the matrix $Q=\left[ \begin{matrix} Q_{1} & \cdots& Q_{n} \end{matrix} \right]$ is the derivative of $\log\text{E}_{Z_{1}}\left( D \right)$ with respect to $\theta$, with its column defined as

$$Q_{i}=\left[ \begin{aligned} \begin{aligned} \begin{aligned} \begin{aligned} \begin{matrix} 1 \\ C_{1,i} \\ C_{2,i} \end{matrix} \\ C_{3,i} \end{aligned} \\ \frac{Z_{1,i}\exp\left( {a_{4}A}_{1,i}+{a_{5}A}_{2,i} \right)}{1+b_{1}Z_{1,i}\exp\left( {a_{4}A}_{1,i}+{a_{5}A}_{2,i} \right)+b_{2}X_{2,i}} \end{aligned} \\ \frac{b_{1}Z_{1,i}A_{1,i}\exp\left( {a_{4}A}_{1,i}+{a_{5}A}_{2,i} \right)}{1+b_{1}Z_{1,i}\exp\left( {a_{4}A}_{1,i}+{a_{5}A}_{2,i} \right)+b_{2}X_{2,i}} \\ \frac{b_{1}Z_{1,i}A_{2,i}\exp\left( {a_{4}A}_{1,i}+{a_{5}A}_{2,i} \right)}{1+b_{1}Z_{1,i}\exp\left( {a_{4}A}_{1,i}+{a_{5}A}_{2,i} \right)+b_{2}X_{2,i}} \end{aligned} \\ \frac{X_{2,i}}{1+b_{1}Z_{1,i}\exp\left( {a_{4}A}_{1,i}+{a_{5}A}_{2,i} \right)+b_{2}X_{2,i}} \end{aligned} \right].$$

The variance of $D$ conditioned on $Z_{1}$ then is

$$\text{Var}\left( D | Z_{1} \right)=\text{E}_{X_{1}|Z_{1}}\left( \text{Var}\left( D | Z_{1},X_{1} \right) \right)+\text{Var}_{X_{1}|Z_{1}}\left( \text{E}\left( D | Z_{1},X_{1} \right) \right)$$

$$=\text{diag}\left( \text{E}_{Z_{1}}\left( D \right) \right)+b_{1}^{2}G \text{Cov}\left( X_{1} | Z_{1} \right)G,$$

where

$$G=\left[ \begin{matrix} t_{1}\exp\left( a+\sum_{j=1}^{J} a_{1,j}C_{1,j} \right) & & \\ & \ddots& \\ & & t_{n}\exp\left( a+\sum_{j=1}^{J} a_{1,j}C_{n,j} \right) \end{matrix} \right].$$

Hence the variance of $S_{Z}$ is

$$\text{Var}\left( S_{Z_{1}} \right)=Q \text{Var}\left( D | Z_{1} \right)Q^{T}$$

$$=I_{Z_{1}}+b_{1}^{2}QG \text{Cov}\left( X_{1} | Z_{1} \right)GQ^{T}$$

# Proof of Theorem 1

**Proof** Let $Z$ be a column vector of $\left\{ Z_{i}, 1\leq i\leq n \right\}$, and define $X,Y$ similarly. Let $1$ be a column vector of 1, $\epsilon_{M}=\left\{ \epsilon_{Mi},1\leq i\leq n \right\}, \epsilon_{A}=\left\{ \epsilon_{Ai},1\leq i\leq n \right\},\epsilon=\left\{ \epsilon_{i},1\leq i\leq n \right\}$.

1. When $\epsilon_{Mi}\equiv1$, the OLS estimator  $\hat{b}_{n}$ is

$$\hat{b}_{n}|\epsilon_{SM},\epsilon_{SA}=\frac{\left( Z-\frac{1}{n}{11}^{T}Z \right)^{T}Y}{\left( Z-\frac{1}{n}{11}^{T}Z \right)^{T}\left( Z-\frac{1}{n}{11}^{T}Z \right)}$$

$$=\frac{\left( Z-\frac{1}{n}{11}^{T}Z \right)^{T}\left( a1+b\epsilon_{SM}Z+\epsilon_{SA}1+\epsilon_{A}+\epsilon\right)}{Z^{T}Z-\frac{1}{n}Z^{T}{11}^{T}Z}$$

$$=b\epsilon_{SM}+\frac{\left( Z-\frac{1}{n}{11}^{T}Z \right)^{T}(\epsilon_{A}+\epsilon)}{Z^{T}Z-\frac{1}{n}Z^{T}{11}^{T}Z}.$$

When

$$\max_{1\leq i\leq n} Z_{i}=_{p}o\left( n^{\frac{1}{2}} \right),$$

we have

$$\max\sqrt{n}\left( \frac{\left( Z-\frac{1}{n}{11}^{T}Z \right)^{T}}{Z^{T}Z-\frac{1}{n}Z^{T}{11}^{T}Z} \right)=_{p}o\left( 1 \right),$$

and when $E\left( Z^{2} \right)<\infty$,

$$n\left( \frac{\left( Z-\frac{1}{n}{11}^{T}Z \right)^{2}}{\left( Z^{T}Z-\frac{1}{n}Z^{T}{11}^{T}Z \right)^{2}} \right)=\frac{n}{Z^{T}Z-\frac{1}{n}Z^{T}{11}^{T}Z}\underset{\to}{a.s.}\frac{1}{\text{Var}\left( Z \right)}.$$

So the second term has asymptotic normal distribution from Lindeberg-Feller Central Limit Theorem (CLT).

Let

$$N_{n}=\frac{\left( Z-\frac{1}{n}{11}^{T}Z \right)^{T}\left( \epsilon_{A}+\epsilon\right)}{Z^{T}Z-\frac{1}{n}Z^{T}{11}^{T}Z}$$

then $N_{n}\perp\epsilon_{SM}$, and thus  $\hat{b}_{n}|\epsilon_{SM}=b\epsilon_{SM}+N_{n},N_{n}\to_{d}N$, where

$$N\sim N\left( 0,\frac{\sigma^{2}+\sigma_{A}^{2}}{n \text{Var}\left( Z \right)} \right).$$

Since

$$\text{E}\left( e^{it\hat{b}} \right)=\text{E}\left( \text{E}\left( e^{it\hat{b}} \right) | \epsilon_{SM} \right)=\text{E}\left( e^{itb\epsilon_{SM}} \right)\text{E}\left( e^{itN_{n}} \right)\to\text{E}\left( e^{it\left( b\epsilon_{SM}+N \right)} \right),$$

applying Continuity theorem gives

$$\hat{b}_{n}\to_{d}b\epsilon_{SM}+N.$$

1. When $\mathrm{Var}\left( \epsilon_{Mi} \right)=\sigma_{M}^{2}>0$,

$$\hat{b}_{n}|\epsilon_{SM},\epsilon_{SA}=b\epsilon_{SM}\frac{Z^{T}\left( \mathrm{diag}\left( Z \right)-\left( \frac{1}{n}Z^{T}1 \right)I \right)\epsilon_{M}}{Z^{T}Z-\frac{1}{n}Z^{T}{11}^{T}Z}+\frac{\left( Z-\frac{1}{n}{11}^{T}Z \right)^{T}\left( \epsilon_{A}+\epsilon\right)}{Z^{T}Z-\frac{1}{n}Z^{T}{11}^{T}Z}.$$

The asymptotic distribution of the second term is known from above. Notice

$$n\left( \frac{Z^{T}\left( \text{diag}\left( Z \right)-\left( \frac{1}{n}Z^{T}1 \right)I \right)}{Z^{T}Z-\frac{1}{n}Z^{T}{11}^{T}Z} \right)^{2}=\frac{\frac{\left( Z^{T}\left( \text{diag}\left( Z \right)-\left( \frac{1}{n}Z^{T}1 \right)I \right) \right)^{2}}{n}}{\frac{\left( Z^{T}Z-\frac{1}{n}Z^{T}{11}^{T}Z \right)^{2}}{n^{2}}}$$

$$=\frac{\sum_{i} Z_{i}^{2}\left( Z_{i}-\bar{Z} \right)^{2}/n}{\left( Z^{T}Z-\frac{1}{n}Z^{T}{11}^{T}Z \right)^{2}/n^{2}}\underset{\to}{a.s.}\frac{\text{E}\left( Z^{2}\left( Z-EZ \right)^{2} \right)}{\text{Var}\left( Z \right)^{2}}.$$

When we have $\max_{1\leq i\leq n} Z_{i}^{2}=_{p}o\left( n^{\frac{1}{2}} \right)$, from Lindeberg-Feller CLT we have

$$\frac{Z^{T}\left( \text{diag}\left( Z \right)-\left( \frac{1}{n}Z^{T}1 \right)I \right)\epsilon_{M}}{Z^{T}Z-\frac{1}{n}Z^{T}{11}^{T}Z}\to_{d}N\left( 1,\frac{\text{E}\left( Z^{2}\left( Z-\text{E}Z \right)^{2} \right)}{n \text{Var}\left( Z \right)^{2}}\sigma_{M}^{2} \right)$$

Using Continuity theorem with characteristic functions as above, we conclude that

$$\hat{b}_{n}\to_{d}b\epsilon_{SM}N_{1}+N_{2}$$

where

$$N_{1}\sim N\left( 1,\frac{\text{E}\left( Z^{2}\left( Z-\text{E}Z \right)^{2} \right)}{n \text{Var}\left( Z \right)^{2}}\sigma_{M}^{2} \right), N_{2}\sim N\left( 0,\frac{\sigma^{2}+\sigma_{A}^{2}}{n \text{Var}\left( Z \right)} \right)$$

$$∎$$
