# Supplementary material for: Correction of confidence intervals in excess relative risk models using Monte Carlo dosimetry systems with shared errors
Source: PLoS One. 2017 Apr 3;12(4):e0174641. doi: 10.1371/journal.pone.0174641 (PMC5378348; doi:10.1371/journal.pone.0174641)
Supplement: S1 Table — The coverage of confidence intervals for all parameters in Eq (1) using different methods in moderate and strong ERR models is given, with DS-U, DS-S, DS-SU, DS-SUP. (DOCX) [file pone.0174641.s002.docx]

**S1 Table. Confidence interval coverage for all model parameters with SUMA dosimetry systems**

| Dosimetry system | Parameter | Naïve Confidence Interval | | Corrected Confidence Interval | |
| --- | --- | --- | --- | --- | --- |
|  |  | Moderate | Strong | Moderate | Strong |
| DS-U | *a*_0_ | .960 (.015, .025) | .953 (.015, .032) | .961 (.014, .025) | .954 (.015, .031) |
|  | *a*_1_ | .955 (.027, .018) | .949 (.035, .016) | .956 (.027, .017) | .952 (.033, .015) |
|  | *a*_2_ | .957 (.024, .019) | .933 (.031, .036) | .957 (.024, .019) | .935 (.030, .035) |
|  | *a*_3_ | .955 (.014, .031) | .951 (.009, .040) | .955 (.014, .031) | .953 (.009, .038) |
|  | ***b*_1_** | **.945 (.054, .001)** | **.906 (.091, .003)** | **.949 (.050, .001)** | **.918 (.079, .003)** |
|  | *a*_4_ | .956 (.020, .024) | .945 (.036, .019) | .956 (.020, .024) | .952 (.033, .015) |
|  | *a*_5_ | .957 (.025, .018) | .939 (.052, .009) | .960 (.023, .017) | .948 (.044, .008) |
|  | *b*_2_ | .938 (.058, .004) | .963 (.021, .016) | .938 (.058, .004) | .963 (.021, .016) |
| DS-S | *a*_0_ | .950 (.014, .036) | .946 (.025, .029) | .950 (.014, .036) | .946 (.025, .029) |
|  | *a*_1_ | .955 (.036, .009) | .952 (.026, .022) | .955 (.036, .009) | .952 (.026, .022) |
|  | *a*_2_ | .952 (.028, .020) | .952 (.029, .019) | .952 (.028, .020) | .952 (.029, .019) |
|  | *a*_3_ | .951 (.022, .027) | .950 (.020, .030) | .951 (.022, .027) | .950 (.020, .030) |
|  | ***b*_1_** | **.663 (.254, .083)** | **.436 (.358, .206)** | **.943 (.039, .018)** | **.947 (.027, .026)** |
|  | *a*_4_ | .945 (.018, .037) | .941 (.027, .032) | .945 (.018, .037) | .941 (.027, .032) |
|  | *a*_5_ | .963 (.018, .019) | .940 (.031, .029) | .963 (.018, .019) | .940 (.031, .029) |
|  | *b*_2_ | .940 (.053, .007) | .954 (.028, .018) | .940 (.053, .007) | .954 (.028, .018) |
| DS-SU | *a*_0_ | .957 (.022, .021) | .946 (.017, .017) | .957 (.022, .021) | .947 (.017, .036) |
|  | *a*_1_ | .956 (.029, .015) | .949 (.025, .026) | .956 (.029, .015) | .953 (.023, .024) |
|  | *a*_2_ | .942 (.026, .032) | .955 (.024, .021) | .943 (.026, .031) | .956 (.024, .020) |
|  | *a*_3_ | .945 (.020, .035) | .937 (.017, .046) | .945 (.020, .035) | .938 (.017, .045) |
|  | ***b*_1_** | **.677 (.251, .072)** | **.448 (.401, .151)** | **.941 (.040, .019)** | **.957 (.025, .018)** |
|  | *a*_4_ | .942 (.032, .026) | .949 (.035, .016) | .944 (.032, .024) | .956 (.029, .015) |
|  | *a*_5_ | .948 (.031, .021) | .947 (.040, .013) | .951 (.029, .020) | .958 (.033, .009) |
|  | *b*_2_ | .954 (.042, .004) | .950 (.035, .015) | .954 (.042, .004) | .950 (.035, .015) |
| DS-SUP | *a*_0_ | .950 (.019, .031) | .943 (.016, .041) | .950 (.019, .031) | .944 (.016, .040) |
|  | *a*_1_ | .949 (.033, .018) | .954 (.028, .018) | .951 (.033, .016) | .955 (.027, .018) |
|  | *a*_2_ | .949 (.031, .020) | .957 (.018, .025) | .950 (.030, .020) | .959 (.018, .023) |
|  | *a*_3_ | .950 (.018, .032) | .936 (.015, .049) | .950 (.018, .032) | .938 (.014, .048) |
|  | ***b*_1_** | **.703 (.230, .067)** | **.467 (.383, .150)** | **.956 (.034, .010)** | **.951 (.037, .012)** |
|  | *a*_4_ | .949 (.024, .027) | .939 (.044, .017) | .952 (.024, .024) | .946 (.038, .016) |
|  | *a*_5_ | .952 (.028, .020) | .933 (.057, .010) | .957 (.025, .018) | .947 (.044, .009) |
|  | *b*_2_ | .938 (.056, .006) | .954 (.034, .012) | .938 (.056, .006) | .954 (.034, .012) |

The coverage of confidence intervals for all parameters in Equation (1) using different methods in moderate and strong ERR models is given, with DS-U, DS-S, DS-SU, DS-SUP.

† Overall coverage (fraction of times the upper bound is below the true value, fraction of times the lower bound is greater than the true value).
