# Supplementary material for: Correction of confidence intervals in excess relative risk models using Monte Carlo dosimetry systems with shared errors
Source: PLoS One. 2017 Apr 3;12(4):e0174641. doi: 10.1371/journal.pone.0174641 (PMC5378348; doi:10.1371/journal.pone.0174641)
Supplement: S2 Table — The coverage of confidence intervals for all parameters in Eq (1) is given. The model was fitted using the true internal dose X1 in both moderate and strong ERR models with 5 dosimetry systems. (DOCX) [file pone.0174641.s003.docx]

**S2 Table. Confidence interval coverage for model parameters fitted with true dose**

| Dosimetry system | Parameter | Moderate model | Strong model |
| --- | --- | --- | --- |
| DS-U | *a*_0_ | .967 (.014, .019)† | .951 (.021, .028) |
|  | *a*_1_ | .954 (.030, .016) | .947 (.033, .020) |
|  | *a*_2_ | .955 (.025, .020) | .937 (.033, .030) |
|  | *a*_3_ | .961 (.017, .022) | .945 (.021, .034) |
|  | ***b*_1_** | ***.*953 (.047, .000)** | **.948 (.044, .008)** |
|  | *a*_4_ | .956 (.015, .029) | .947 (.027, .026) |
|  | *a*_5_ | .950 (.023, .027) | .949 (.028, .023) |
|  | *b*_2_ | .940 (.056, .004) | .958 (.028, .014) |
| DS-S | *a*_0_ | .950 (.014, .036) | .946 (.025, .029) |
|  | *a*_1_ | .955 (.036, .009) | .952 (.026, .022) |
|  | *a*_2_ | .952 (.028, .020) | .952 (.029, .019) |
|  | *a*_3_ | .951 (.022, .027) | .950 (.020, .030) |
|  | ***b*_1_** | **.932 (.068, .000)** | **.943 (.041, .016)** |
|  | *a*_4_ | .945 (.018, .037) | .941 (.027, .032) |
|  | *a*_5_ | .963 (.018, .019) | .940 (.031, .029) |
|  | *b*_2_ | .940 (.053, .007) | .954 (.028, .018) |
| DS-SU | *a*_0_ | .953 (.023, .024) | .949 (.021, .030) |
|  | *a*_1_ | .949 (.031, .020) | .954 (.022, .024) |
|  | *a*_2_ | .949 (.022, .029) | .952 (.023, .025) |
|  | *a*_3_ | .949 (.021, .030) | .951 (.022, .027) |
|  | ***b*_1_** | **.936 (.057, .007)** | **.942 (.044, .014)** |
|  | *a*_4_ | .943 (.025, .032) | .965 (.018, .017) |
|  | *a*_5_ | .949 (.027, .024) | .946 (.019, .035) |
|  | *b*_2_ | .951 (.045, .004) | .947 (.038, .015) |
| DS-SUP | *a*_0_ | .956 (.017, .027) | .953 (.021, .026) |
|  | *a*_1_ | .949 (.034, .017) | .956 (.025, .019) |
|  | *a*_2_ | .950 (.030, .020) | .948 (.021, .031) |
|  | *a*_3_ | .951 (.021, .028) | .949 (.020, .031) |
|  | ***b*_1_** | **.938 (.061, .001)** | **.949 (.041, .010)** |
|  | *a*_4_ | .960 (.017, .023) | .943 (.026, .031) |
|  | *a*_5_ | .960 (.014, .026) | .950 (.023, .027) |
|  | *b*_2_ | .937 (.058, .005) | .950 (.039, .011) |
| MWDS-2013 | *a*_0_ | .966 (.013, .021) | .956 (.022, .022) |
|  | *a*_1_ | .960 (.024, .016) | .947 (.027, .026) |
|  | *a*_2_ | .965 (.013, .022) | .956 (.020, .024) |
|  | *a*_3_ | .943 (.023, .034) | .953 (.021, .026) |
|  | ***b*_1_** | **.962 (.037, .001)** | **.950 (.035, .015)** |
|  | *a*_4_ | .959 (.019, .022) | .960 (.019, .021) |
|  | *a*_5_ | .959 (.021, .020) | .953 (.026, .021) |
|  | *b*_2_ | .926 (.070, .004) | .952 (.036, .012) |

The coverage of confidence intervals for all parameters in Equation (1) is given. The model was fitted using the true internal dose *X*_1_ in both moderate and strong ERR models with 5 dosimetry systems.

† Overall coverage (fraction of times the upper bound is below the true value, fraction of times the lower bound is greater than the true value).
